# High-throughput approaches for the identification of ribosome heterogeneity

**DOI:** 10.1098/rstb.2023.0381

**Published:** 2025-03-06

**Authors:** Edwin S. Kyei-Baffour, Qi Chang Lin, Ferhat Alkan, William J. Faller

**Affiliations:** ^1^ Division of Oncogenomics, The Netherlands Cancer Institute, Amsterdam 1066CX, The Netherlands; ^2^ Department of Neurology and Neurosurgery, University Medical Center Utrecht Brain Center, Utrecht University, Utrecht, The Netherlands

**Keywords:** ribosome heterogeneity, rRNA, ribosomal proteins, high-throughput techniques

## Abstract

Recent advances in the fields of RNA translation and ribosome biology have demonstrated the heterogeneous nature of ribosomes. This manifests not only across different cellular contexts but also within the same cell. Such variations in ribosomal composition, be it in ribosomal RNAs or proteins, can significantly influence cellular processes and responses by altering the mRNAs being translated or the dynamics of ribosomes during the translation process. Therefore, identifying this heterogeneity is crucial for unravelling the complexity of gene expression across different fields of biology. Here we provide an overview of recent advances in high-throughput techniques for identifying ribosomal heterogeneity. We cover methodologies for probing both rRNA and protein components of the ribosome and encompass the most recent next-generation sequencing and computational analyses, as well as a diverse array of mass spectrometry techniques.

This article is part of the discussion meeting issue ‘Ribosome diversity and its impact on protein synthesis, development and disease’.

## 1. Introduction

The ribosome, a macromolecular complex central to all life on Earth, was first discovered in 1955 by George E. Palade. He found small free-floating and endoplasmic reticulum (ER)-bound particles in the cytoplasm of mammalian and avian cells [1]. At the time Palade postulated that the ribosomes may be heterogeneous [2], based on the observation that the particles he observed had noticeable differences in morphology, physiochemistry and metabolic levels [3]. Francis Crick developed the ‘one gene-one ribosome-one protein’ hypothesis, implying that for every gene encoded in the genome, a distinct ribosome would exist to create the gene-encoded protein [4]. However, the idea of heterogeneous ribosomes was largely discarded after the discovery of mRNA [5], and the prevailing view of the ribosome was of a homogenous macromolecular complex without any variability and potential for specialization.

Over the last 30 years, however, evidence supporting the existence of heterogeneous ribosomes has gradually accumulated [2]. This began with the finding that mutations in ribosomal proteins (RPs) and sequence variations in rRNAs resulted in phenotypic differences [6–8], and it later became clear that RP paralogues are expressed in a tissue-specific manner, suggesting differential composition of ribosomes for each tissue [9]. Additionally, haploinsufficiency or mutation of genes encoding RPs has also been shown to have phenotypic effects that differ in a tissue-dependent manner, known as ribosomopathies [10]. Diamond–Blackfan anaemia is perhaps the most famous example of such a ribosomopathy, where specific mutations in RP-encoding genes can be linked to a diverse set of symptoms found in these patients [11]. Recently, advances in molecular tools allowed for the detection of RPs that show variability in ribosome incorporation, such as RPL10A/uL1, RPS25/eS25 and RPL38/eL38, which were each associated with mRNAs involved in different cellular and metabolic processes [12]. Other RPs (RPL11/uL5 and RPL5/uL18) and modifications (2′-*O*-methylation (2′-*O*-me) [13] and pseudouridylation (Ψ) [14]) have been implicated in ribosomal heterogeneity, and specialized ribosomes such as the onco-ribosome and immuno-ribosomes have been hypothesized [15–17]. Ultimately, the field of ribosome heterogeneity was initiated and continues to be investigated.

Our current understanding of ribosome heterogeneity is still in its infancy. However, there are increasing technological advances in wet lab- and dry lab-based techniques to study it, including sequencing techniques, mass spectrometry (MS) techniques and algorithms predicting ribosome heterogeneity [18]. The increase in tools comes with the difficulty in selecting suitable tools for specific research questions. For example, methods developed with one specific goal in mind may not be suitable for another [19]. In this review, we try to illustrate some of the common methods used in the field to detect and study ribosome heterogeneity, focusing mainly on the bioinformatic tools or approaches used.

## 2. rRNA-based ribosome heterogeneity

In both prokaryotic and eukaryotic cells, rRNA is transcribed from segments of the genome called ribosomal DNA (rDNA) [20,21]. In eukaryotes, these segments are primarily located in the nucleolus organizer regions of multiple chromosomes, and are organized as tandem arrays. Due to the highly repetitive nature of eukaryotic rDNA, substantial levels of insertion and deletion (indels; due to error-prone recombination) are present [22], but this is not the case in prokaryotes that tend to have their rDNA operons dispersed across the entire genome [23].

Although the role of rRNAs in defining distinct ribosome populations is not as established as it is in RPs, advances in sequencing technologies have recently garnered increased attention for rRNAs [18]. Differences in rRNA variants [24] and modifications [25] in actively translating ribosomes have all been shown to be detectable in live cells and present a rich potential source of ribosome heterogeneity.

### (a) rRNA variants

rRNA variation in ribosomes has always (rightly) been considered tricky to detect. Humans are thought to have 200–400 copies of rDNA [26], and heterogeneity between these has been recognized for decades [27]. Despite this variation, rDNA copies have extremely high sequence homology, and as a result, they are usually excluded from sequencing efforts [28]. This has resulted in an unfortunate but understandable lack of reference sequences. However, the advent of long-read sequencing technologies, such as Oxford Nanopore Technologies (ONT) sequencing, has led to the complete sequencing of the human genome. With this approach, the rDNA reads are used to construct sparse de Bruijn graphs [29,30], and using walks across the graph, the sequenced nanopore reads are de novo aligned. Ultimately, similar sequences were clustered to form an rDNA unit, and a consensus sequence was built after polishing with PacificBio HiFi sequencing [31].

**Figure 1.**
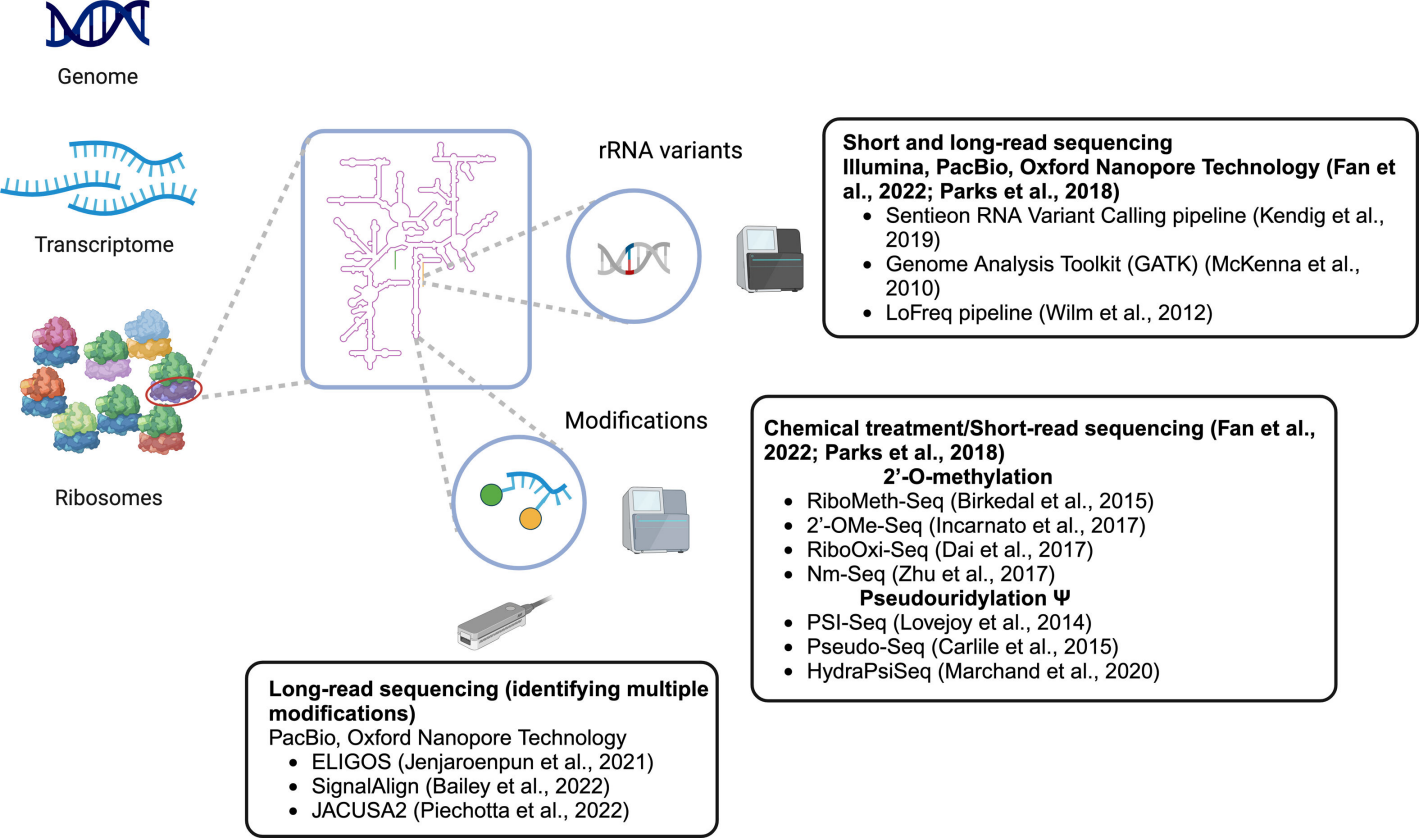
Identifying rRNA variants and modifications.

A recent study identified 3791 variants in human rRNA genes, which were found in both gene coding and intergenic regions, but largely absent from both rRNA modification sites and RP interaction sites [32]. In identifying different variants of rRNA, the Sentieon RNA Variant Calling pipeline, which is analogous to the Genome Analysis Toolkit (GATK) was used (figure 1) [33,34]. GATK uses a Bayesian approach that calls a variant given mapped reads at a locus. The base-call quality scores at each locus are used to determine the conditional probability of a base over all the genotypes. This is used to determine the genotype based on the mapped reads at a locus. Duplicates are removed from mapped reads, and unique reads are remapped to eliminate biases introduced in regions with insertions and deletions. Base scores are recalibrated using a base-error model to eliminate base quality bias introduced by the sequencer. Variants are counted and differentially analysed for their expression between samples [24,32].

Others have shown that rRNA variants are differentially expressed across mouse tissues and organs and are incorporated into active ribosomes using the LoFreq pipeline [24]. The LoFreq pipeline has also been used for analysing rRNA variants, identifying the expression of different variants and identified in translating ribosomes (figure 1) [24,35]. LoFreq models each base as a Bernoulli trial using the Phred quality score. The number of variants at a locus is modelled as a Poisson-binomial distribution where the probability of observing variants at a locus is recursively determined through the sum of the tail of the probability mass function (pmf). The recursion is optimized by eliminating intermediate pmfs that do not affect the *p*-value determined. To call variants, the quality of mapped reads is modelled similarly to GATK and is fed as an input to the LoFreq pipeline.

The tools described here have been used to identify variations in rRNA sequences; however, their effect on ribosome function is yet to be demonstrated [28]. Though LoFreq and GATK are sensitive and minimize false positives, benchmarking of variant callers does not account for tandem repeats and segmental duplications observed in rDNA [28]. Therefore, these tools require additional benchmarking specifically on rRNA regions to assess the quality of rRNA variants identified.

### (b) rRNA modifications

**Figure 2.**
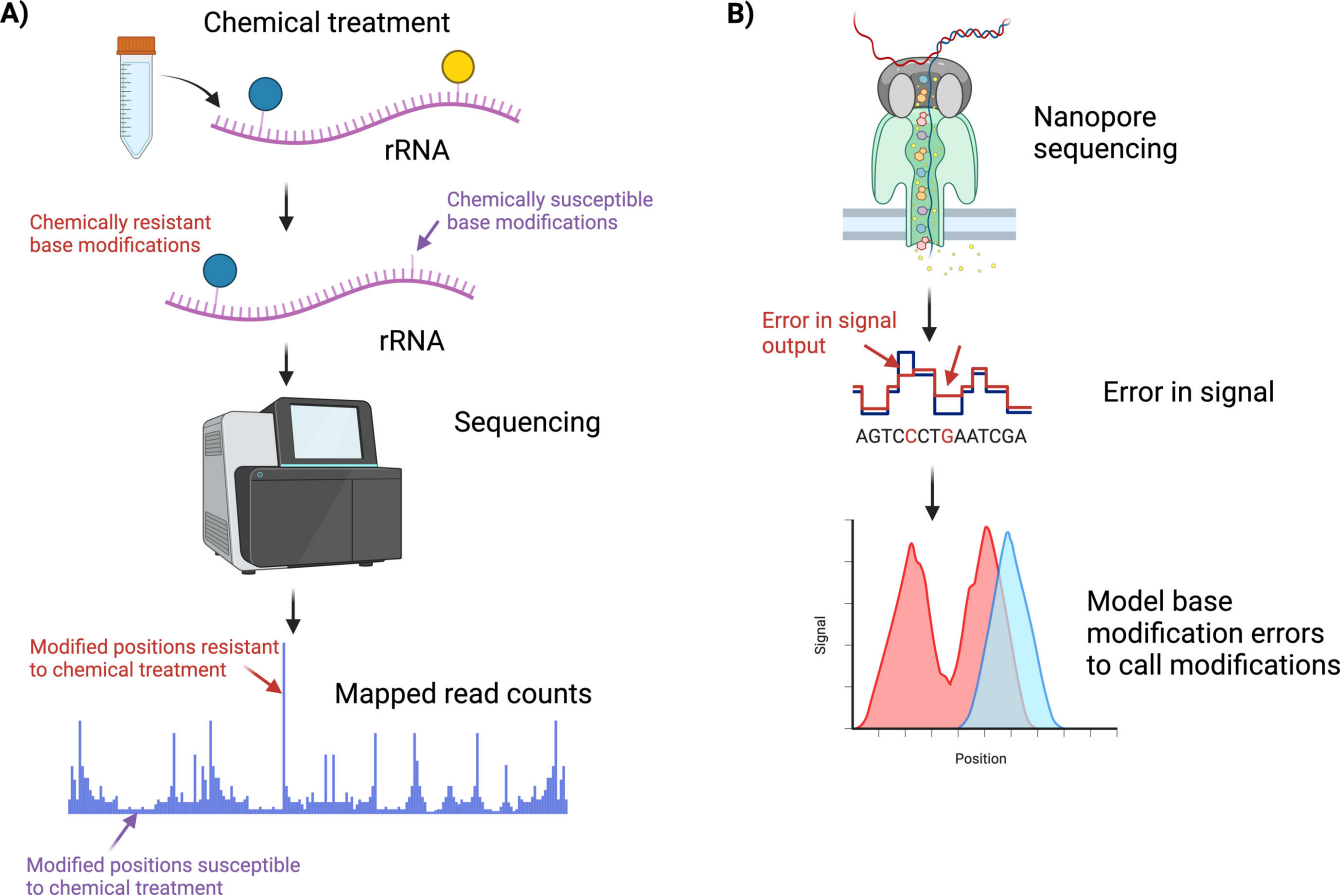
Common approaches to identifying modified rRNA positions. (*A*) Chemical-based detection methods relying on the chemical characteristics of the modification are identified through sequencing of chemically treated rRNA. (*B*) Long-read-based methods model the error rates in calling bases to identify modified bases of the rRNA.

Different post-transcriptional modifications are found throughout rRNAs; however, the large majority of these are 2′-*O*-me and pseudouridylation (Ψ) [18]. Similar to variant identification, as sequencing has become more accurate and cheaper, specialized techniques to analyse the epitranscriptome have also started to appear [36], primarily focused on 2′-*O*-me and Ψ [18]. Tools for studying these common variations can be split into chemical treatments followed by sequencing and long-read sequencing methods (figures 1 and 2).

#### (i) 2′-*O*-methylation

RiboMeth-Seq is one of the chemical-based techniques for detecting 2′-*O*-me modification (figures 1 and 2). The method makes use of the fact that the 2′-*O*-me modification makes the neighbouring phosphodiester bond resistant to alkaline degradation. After partial alkaline hydrolysis, short fragments of 20−40 nucleotides remain from the original rRNAs. These fragments are then sequenced as inserts and aligned to the reference. During the alignment, the first and the last nucleotide of the insert is also recorded. Due to the resistance to alkaline degradation, the methylated base on the 5′ read-ends will not be aligned properly in the mapping step. This will eventually result in a gap in the alignment, where the 5′ read-ends shift one position upstream [37]. Partially digested rRNA produces fragments that are sequenced and mapped to a reference sequence. Since the expectation is to observe less digestion at methylated bases, counting of the mapped reads is only done at the end of the reads. The 5′, 3′ and a combined 5′ and 3′ count are used to generate a RiboMeth-Seq score. This score is calculated by comparing the number of reads at a position to the average number of reads and the standard deviation 12 positions (six on each side) around the base of interest. Positions are identified as methylated when they exceed the optimal threshold set with Matthew’s correlation coefficient [37,38].

The 2′-*O*Me-Seq uses a very different approach, in which the reverse transcriptase (RT) in polymerase chain reaction (PCR) is halted due to low dNTP concentrations in the PCR cocktail at a site with a 2′-*O*-me modification [39]. The short cDNA generated by the differential processivity of the RTs can then be sequenced. By comparing its sequencing alignment with one that contained a normal concentration of dNTP in the PCR cocktail, one can find the relative quantification of the 2′-*O*-me levels in the processed sample [40].

Finally, RiboOxi-Seq and Nm-Seq can also be used in the detection of 2′-*O*-me modifications [41]. Both methods exploit the periodate cleavage resistance of the 2′-*O*-me modification. First, rRNAs are fragmented chemically or by nucleases. Afterwards, the fragmented rRNAs are subjected to periodate oxidation, repeatedly cleaving bases from the 3′ end. Fragments with a 2′-*O*-me 3′-terminus are resistant to periodate cleavage and form dialdehydes structures, leaving 3′ 2′-*O*-me containing fragments as the only candidates for 3′-adapter ligation. In analysing the sequenced data to identify 2′-*O*-me, 3′ ends are counted and normalized. The normalized counts are either compared to the background by finding the difference [42] or used as input for the DESEQ2 pipeline [43] to determine enrichment and statistical significance [44]. Thus, sequenced reads will only contain information about the fragments with a 2′-*O*-me 3′-terminus, which enables the detection of the modification [44,45].

RiboMeth-Seq, compared to the other methods, lacks specificity due to the random fragmentation and detection being biased towards more abundant starting RNAs [46]. The 2′-*O*Me-Seq, RiboOxi-Seq and Nm-Seq improve specificity and sensitivity by targeting RNAs with this modification. However, they do not determine the absolute quantification of modification, and 2′-*O*Me-Seq requires sequencing samples with high and low concentrations of nucleotides [40]. RiboOxi-Seq and Nm-Seq are more efficient in detecting modifications at the 3′ end, and their ability to detect modification decreases the closer to the 5′ end it is. Additionally, they are both limited in detecting modifications adjacent to each other. Nm-Seq sacrifices stoichiometric information for sensitivity while RiboOxi-Seq is biased in generating the sequencing library [42,44].

#### (ii) Pseudouridylation (Ψ)

To detect Ψ modifications in rRNAs, multiple sequencing options are also available. Pseudouridine site identification sequencing (PSI-Seq) makes use of 1-cyclohexyl-(2-morpholinoethyl) carbodiimide metho-p-toluene sulfonate (CMCT) in the preparation of the sequencing library [47]. When CMCT is added to RNA with a Ψ modification, a carbodiimide (CMC) adduct will form at the N1 and N3 of Ψ. Upon treatment with a mild alkali, CMC will be removed from N1, but the CMC adduct will stay on N3 [48]. This will then block reverse transcription, resulting in shorter transcripts. Following library preparation and sequencing, the base at the 5′-end of the sequencing read can be used to map Ψ modifications [47].

Another technique, Pseudo-Seq [49], is highly similar to PSI-Seq. Although small variations exist in the library preparation protocols, the primary difference lies in the data analysis. PSI-Seq uses a regression analysis between sequencing data from CMCT-treated and CMCT non-treated samples to differentiate locations of the Ψ modification, while Pseudo-Seq used the read counts of bases located next to a uridine to computationally calculate peaks to call locations with a Ψ modification [50].

The two methods presented, PSI-Seq [47] and Pseudo-Seq [49], both require CMCT in the sequencing protocols, which generates a lot of sequencing noise. HydraPsiSeq is a method similar to RiboMeth-Seq (discussed above), which may overcome this issue [51]. Instead of CMCT, a combination of hydrazine and aniline is used. This cleaves the RNA at uridine; however, Ψ protects the base, therefore making it identifiable via sequencing.

Other post-transcriptional modifications, such as m6A, m7G, m1A and m5C, are also found to a lesser extent in rRNAs [18,52]. Although they may well play a role in rRNA-induced ribosome heterogeneity, the evidence is currently scarce, so they will not be described here.

### (c) Long-read sequencing technologies

Most current methods to detect rRNA modifications are based on chemical treatment of the RNA prior to sequencing. However, this is known to produce noisy data and introduce biases to the results, something that can be overcome using long-read sequencing technologies, such as ONT (figure 2). Different ONT approaches have been used to identify rRNA modifications including epitranscriptional landscape inferring from glitches of ONT signals (ELIGOS) [53], SignalAlign [54] and JACUSA [55,56], the pros and cons of which will be described below.

In SignalAlign, a variable-order hidden Markov model is used to determine modified positions by training on modifications at known sites using the distribution of k-mers [54]. Every round of training generates a Gaussian distribution of k-mers around the modified position with the mean and standard deviation of the distribution corresponding to the median and the median absolute deviation of the empirical k-mer distribution. The posterior probabilities of the trained model are transformed into the probability of a position being modified [54]. Although SignalAlign can be trained to identify multiple modifications, it requires prior knowledge of where these modifications occur.

ELIGOS employs positions with ambiguous base calls using an RNA background error model (rBEM) or a reference sequence for comparison to the native sequenced RNA [53]. In determining modified bases, the error of specific bases (ESB) is calculated for each base with at least 50 mapped reads. The ESB is the frequency of errors in the sequenced RNA compared to the reference, and it is calculated at a five nucleotide length (the number of bases in the nanopore at a time) and shifted across the reference sequence one base at a time. Fisher’s exact test with Benjamini–Hogberg *p*-value correction is used to determine significantly different per cent ESB between the native RNA and the rBEM or reference sequence. The test is done for the following three scenarios: the middle position, the three residues in the middle and all the five residues. The highest odds ratio from Fisher’s exact test is recommended, indicating modifications in one or neighbouring positions. This was applied to modifications including m6A, Ψ, m7G, m1A, Ino, hm5C, 5moU, f5C and m5C [53]. ELIGOS does not work with replicate samples in its pipeline.

Similar to ELIGOS, JACUSA also uses base-calling errors and pairwise comparisons to determine RNA modifications [55]. It models the base call and the quality using the Dirichlet-multinomial distribution where the probability of the occurrence of each base is estimated using an empirical Bayesian approach. This is extended to multiple samples using a maximum likelihood estimation to determine the probability of a base with an included error term to account for sequencing errors. The likelihood ratio test is used to determine significant changes in the distribution of base counts per position between conditions [55].

In a subsequent revision of JACUSA (JACUSA2), a beta-binomial distribution was used to build a general framework for calling base changes [56]. Indel events were also modelled using the same distribution and statistical test, where the occurrence of an indel is determined by the number of reads spanning the position with or without an indel. Using the mismatch, insertion and deletion calls as a feature set different JACUSA2 scores are calculated to determine rRNA modification [56]. In some cases, the local outlier factor is used to determine outlier positions inferring the presence of a modification [57].

Generally, ONT suffers from relatively higher error rates compared to short-read methods. Additionally, the cost and requirement for high levels of starting material could impede the use of these tools which are based on the technology [46]. Though JACUSA2 can rapidly identify novel modifications [56], other tools, like EpiNano [58] and ELIGIOS [53], perform better in identifying different modifications and the occurrence of modifications in different base contexts (A, G, C, U) [56]. EpiNano builds a machine-learning model from features of mapped reads and has been demonstrated for m6A modifications in mRNA [58]. Although ONT has many advantages, it does struggle with correctly predicting modifications with as low as 32% recovery rate in m6A modifications [58]. Identifying modified sites is affected by different factors including different base contexts, read coverage, technical replicates and the type of modification [56].

## 3. Protein-based ribosome heterogeneity

**Figure 3.**
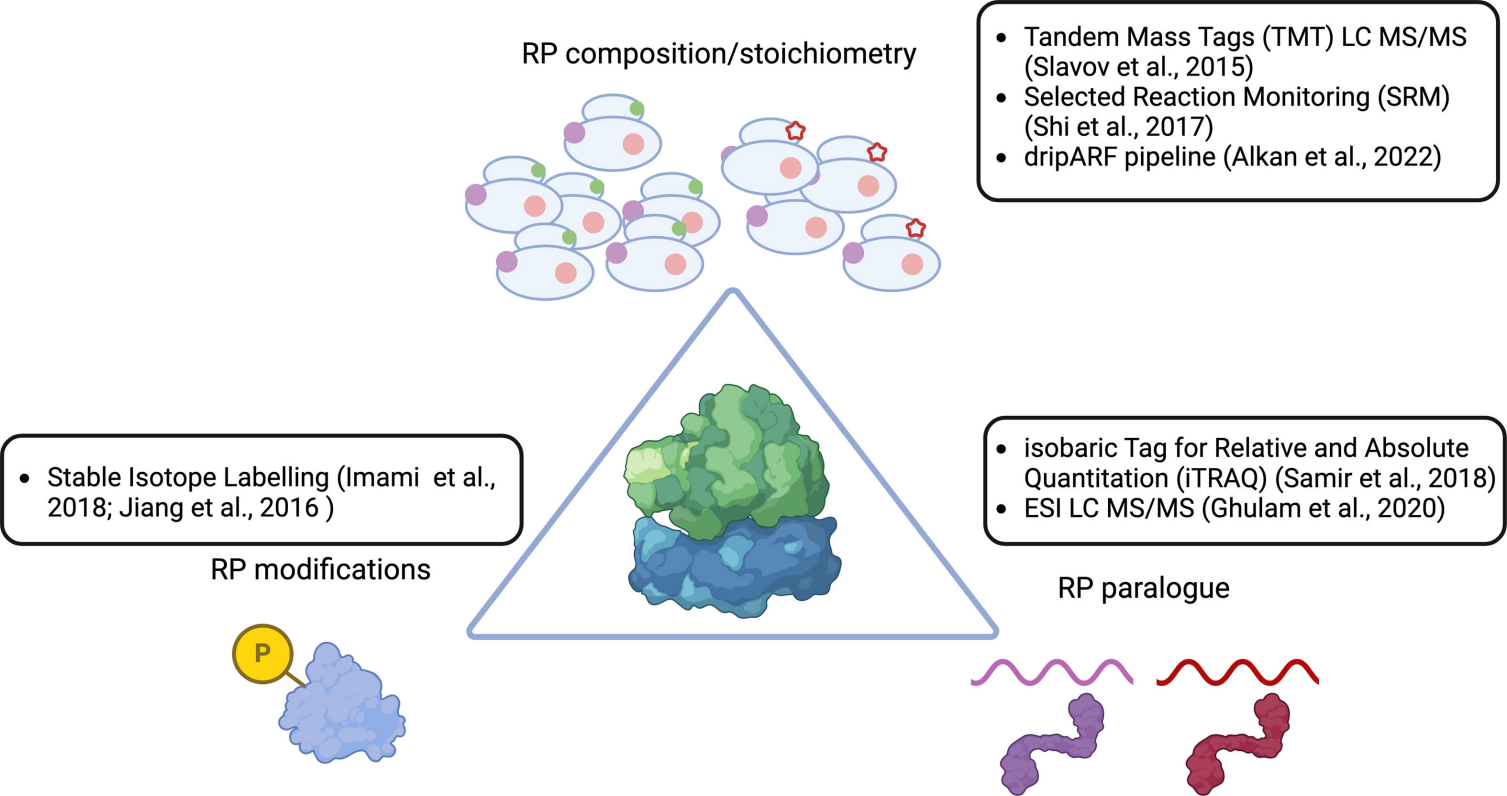
High-throughput methods for identifying RP-mediated ribosomal heterogeneity.

Together with rRNAs, RPs make up the remainder of a ribosome. In the past decades, a number of techniques have been developed to study RP stoichiometry and RP modifications, variations in either of which can potentially lead to a diverse set of ribosomes [59]. The majority of these are MS based, and these are also the most suitable for high-throughput analysis (figure 3). While other techniques are available (cryoEM, for example), these tend to be more suitable for low-throughput analysis, and as a result, we will not include these in our discussion.

### (a) Ribosomal protein composition/stoichiometry

One of the most powerful current tools enabling the detection of RP composition is MS. In particular, quantitative methods developed on the liquid chromatography platform coupled to tandem MS (LC–MS/MS) tend to dominate the field [59]. This can be further classified into discovery MS (label-free), isotopic labelling MS (isobaric labelled or metabolic labelled) and targeted MS (selected reaction monitoring (SRM) or parallel reaction monitoring (PRM)) [60].

Discovery MS is the most commonly used; however, it has its drawbacks. Discovery or untargeted MS without labelling has a lower reproducibility compared to labelled methods like reaction monitoring techniques, does not allow for sample multiplexing and cannot be used for absolute quantification of proteins [59]. Peptides can be absent among replicates, which affects reproducibility, and quantification can be affected by technical artefacts such as poor ion resolution [61].

MS methods with isotopic labels, on the other hand, can be used for absolute and relative quantification of RPs while allowing for sample multiplexing. Isobaric labelling approaches, like tandem mass tags (TMT) [62] and isobaric tags for relative and absolute quantitation (iTRAQ) [63], rely on the addition of a tag to multiple individually digested samples and then mixing the multiple samples at equimolar ratios before LC–MS/MS analysis [60].

This approach was used by Slavov *et al*. [64] in 2015 to provide direct evidence for differences in RP stoichiometry between ribosomes. In the study, monosomes and polysomes from mouse embryonic stem cells and yeast were isolated by velocity sedimentation in sucrose gradients and thereafter digested into peptides. These peptides were then labelled per individual sucrose fraction with TMT and quantified with tandem MS. To make the quantification more robust and consistent, two different digestion enzymes were used (trypsin and lys-C), and unique peptides from each RP were used for comparison. In place of the usually computed intensity-based absolute quantification scores (intensity divided by the number of theoretical peptides generated by digestion) [65], the relative MS2 intensities of peptides unique to each RP were determined. The median intensity was used to represent the relative amount of each RP. Relative quantification prevents the influence of external factors (including protein digestion efficiency and peptide ionization) on the quantification of peptides/proteins. In the final results, the authors could detect differential RP levels across the monosome and polysome fractions, indicating the presence of differential RP stoichiometry in some RPs [64].

Similar evidence has also been provided using targeted MS, specifically, SRM-based proteomics [12]. Like isotopic labelling methods, SRM makes use of external labels before analysis with MS. In using SRM, after proteome digestion of the sample, a known amount of synthetic heavy isotope-labelled peptides is added to the sample, and they are analysed in the mass spectrometer. Peptides are fragmented, generating transition ions whose retention time and signal intensity can be measured. By using the heavy peptides as a reference point, ratios between the transition ions’ fragment peaks of the endogenous peptides from the original sample and the heavy peptides can be calculated. With these ratios, absolute peptide amounts can be correctly acquired for the endogenous proteins in the samples [66]. Shi *et al*. [12] used this method to show that translating polysomes contain differential stoichiometry of RPs compared to monosomes. To quantify each RP, *in silico* trypsin-digested peptides with high signal intensities were selected for each RP, and the top four to six precursors were selected for each peptide after mixing light- and heavy-labelled peptides. The ratio of the transition fragment peak integrals of light to heavy peptides was scaled by the known amount of the heavy peptide introduced into the purified proteins, and the median of the selected peptides was used to determine the absolute quantity of each RP [12].

RPs are small proteins, and finding proper synthetic peptides to act as comparison material to the peptides originating from the relatively small endogenous RPs is difficult, and the cost of synthesizing these heavy peptides is prohibitive [12]. Additionally, SRM monitors ion transitions of each peptide in series, limiting the resolution of the actual peptide. PRM [67] is an associated technology that could potentially solve this problem. The difference is in the third quadrupole of the triple quadrupole set-up, where it is replaced by an Orbitrap or TOF analyser. The technique allows for the analysis of all ion transitions of the product, allowing for high-resolution acquisition [67].

Finally, RP stoichiometry can also be detected using sequencing data on rRNAs. Ribosome profiling is being used more and more often, as the technique allows for the direct measurement of translation efficiency by scrutinizing positional and quantitative information from ribosomal-protected RNA fragments [68–70]. As this methodology necessitates the enrichment of the ribosome, a large volume of rRNA-mapped reads is also generated. We have shown that this data can be used to detect RP composition in a ribosomal pool [71]. The tool, dripARF, is based on the idea that different RP composition of the ribosomes causes differential rRNA digestion in the Ribo-Seq protocol. Using an RP–rRNA proximity matrix (which is the molecular distances between the two in a resolved ribosome structure), rRNA proximity sets are generated for each specific RP. To predict RPs that are heterogeneous across different samples, differential rRNA fragment analysis is done between the different samples for every position across the rRNAs using DESEQ2 [43]. Normalized enrichment score (RPSEA_NES), *z*-transformed randomized NES (RPSEA_NES.randZ) and overrepresentation score are determined to find the actual heterogeneous candidate RPs between the samples [71].

dripARF facilitates fast hypothesis testing of ribosome heterogeneity and large-scale analysis of Ribo-Seq datasets to identify possible RP candidates for ribosomal heterogeneity. However, it is limited by the ribosome structure, the protocol used and the inability to determine the directionality of the changes observed. Protocol variations concerning RNase digestion and rRNA depletion are minimized by restricting comparisons to specific studies [71]. Also, the effect of different protocols on the distribution of reads can be studied across large datasets to highlight RPs that are likely sensitive to specific protocols. Considering these limitations, users are advised to confirm dripARF predictions using other targeted experimental approaches [71].

### (b) Ribosomal protein paralogues

Several studies have used the isobaric labelling method, iTRAQ, in order to assess differences in RP paralogue incorporation. Samir *et al.* determined the differential incorporation of RPL8A (eL8A) and RPL8B (eL8B) in yeast when grown in different carbon sources [72]. Like other isobaric labelling methods mentioned above, iTRAQ tags are used to label the proteins in the sample and the intensity of the reporter ions in the fragmentation spectra is used in quantification [73]. Quantification of each paralogue was determined using unique peptides that matched only one of the paralogues. Further validation of the results of the iTRAQ quantification was done using SRM [72].

In another study of the effects of cellular stress on ribosome composition in yeast, Ghulam *et al.* [74] identified the differential incorporation of major and minor paralogues (duplicated RPs) into ribosomes. Using untargeted electrospray ionization LC–MS/MS, major and minor paralogues were identified by integrating spectra of unique peptides that differentiate duplicated RPs [74]. The intensity of duplicated RPs were normalized across samples using peptides unique to RPs, to enable comparison across samples, and a ratio between the paralogues was computed to assess major and minor RP paralogues.

The need for validation in iTRAQ quantification serves as a drawback for the quantification of RP paralogues. However, iTRAQ provides a better quantitative measure than untargeted MS approaches as highlighted earlier in this review.

### (c) Ribosomal protein modifications

Perhaps the richest potential source of ribosome heterogeneity is in RP post-translational modifications. These modifications include phosphorylation and ubiquitination. Phosphorylation of eS6 is downstream of mTORC1 and has been implicated in neuronal activation and in the initiation of pancreatic cancer [75,76]. Ubiquitination of specific residues in some small subunit RPs (uS5 and uS3) is involved in unfolded protein response [76,77]. Other modifications of RPs include acetylation, methylation and methylthiolation; however, their role in driving ribosome heterogeneity is currently unclear [78].

These modifications can be detected using MS methods already discussed [59], with the addition of a step to enrich phosphopeptides. This is generally done using TiO_2_-based metal-oxide affinity chromatography (MOAC), with immobilized-metal affinity chromatography (IMAC) or with antibody-based enrichment [79]. This enrichment is also part of phosphorylation analysis using stable isotope labelling of amino acids in cell culture (SILAC) [80], which has been applied to the detection of RP phosphorylation in the ribosome [81,82]. SILAC is based on the metabolic incorporation of light or heavy isotope labels into proteins *in vivo*. These are then mixed and digested before LC–MS/MS analysis. Both studies identify phosphorylated residues by determining the probability of observing phosphorylated peptide sequences compared to a theoretical distribution of different phosphorylation combinations. A cumulative binomial distribution function is used to determine the score for different peptide ions that are selected in a 100 m/z unit window and the probability of a phosphorylated ion [83,84].

The detection of phosphorylation events does come with challenges, however. Phosphorylation sites are rare relative to the total proteome and are highly dynamic, meaning that relevant peptides are often of very low abundance. This is partially addressed by the enrichment for phospho-peptides and mitigated by the fact that RPs are some of the most abundant proteins in the cell. On the other hand, RPs tend to have a lower number of tryptic digestion sites, so the use of alternative digestion enzymes may be advantageous [64].

The identification and quantification of RP modifications can be accomplished (figure 3), as can the identification of interacting factors. Due to the high number of phosphorylation sites [85], it is critical to separate those that play a function role in translation. Additionally, it would also be interesting to apply a non-digestion-based MS approach (so-called top–down MS) to intact ribosomes, to study structural differences caused by the diversity of RP modifications and to see if these structural differences influence ribosomal function [86].

## 4. Discussion and conclusion

As illustrated, different methods exist to detect and study ribosome heterogeneity summarized in table 1. On one end, you have several specialized sequencing methods and dedicated computational tools to detect rRNA stoichiometry, rRNA composition and rRNA modifications. On the other end, you have a diverse set of MS-based techniques to detect and study RP stoichiometry, RP composition and RP modifications. It is safe to say that researchers in the field of ribosome heterogeneity are not limited in tools as discussed here; however, these options have their drawbacks. Follow-up experiments or additional data-handling steps need to be carried out after detection.

**Table 1.** Strengths and weakness of high-throughput methods in detecting and quantifying ribosomal heterogeneity.

Heterogeneity type	Modification/approach	Tools^a^	Pros	Cons	Ref
rRNA variants	DNA sequencing	Sentieon RNA variant calling pipeline (GATK)	sensitive and minimizes false positives	does not account for tandem repeats and segmental duplications in rDNA	[24,32,35]
		LoFreq pipeline			
rRNA modifications	2′-O-methylation	RiboMeth-Seq	more efficient in detecting modifications at the 3′ end	lacks specificity due to random fragmentation; biased towards more abundant starting RNAs	[37]
		2′-OMe-Seq	improved specificity and sensitivity	cannot determine absolute quantification of modification; requires sequencing samples with high and low concentrations of nucleotides	[39]
		RiboOxi-Seq	improved specificity and sensitivity	limited in detecting adjacent modifications; decreased ability to detect modification closer to 5′ end; biased in generating sequencing library	[44,45]
		Nm-Seq	more efficient in detecting modifications at the 3′ end; provides stoichiometric information	limited in detecting adjacent modifications; reduced sensitivity; decreased ability to detect modification closer to 5′ end	
	pseudouridylation (Ψ)	PSI-Seq	provides single-base resolution; can identify novel modifications	high sequencing noise; biased towards sites with more reads	[47]
		Pseudo-Seq			[49]
	long-read sequencing technologies	SignalAlign	keeps long-range relationship between modifications	cannot identify novel modifications; relatively higher error rates; affected by base contexts	[54]
		ELIGOS	identifies different modifications, and the occurrence of modifications in different base contexts (A, G, C, U)	relatively higher error rates	[53]
		JAVA framework for accurate SNV assessment (JACUSA/JACUSA2)	rapidly identify novel modifications	relatively higher error rates; affected by base contexts	[55,56]
		EpiNano	identifying different modifications; not affected by different base contexts	cannot identify all modifications (low recovery rate)	[58]
RP composition/stoichiometry	discovery or untargeted MS		high-throughput	lower reproducibility compared to labelled methods; no sample multiplexing; no absolute quantification of proteins; missing peptides in some replicates; quantification can be affected by technical artefacts	[59]
RP paralogues				requires the identification of unique peptides restricted to only one paralogue	[74]
RP composition/stoichiometry	MS with isotopic labels	TMT	high-throughput; absolute and relative quantification of RPs; sample multiplexing		[64]
RP paralogues		iTRAQ	better quantitative measure	finding proper synthetic peptides; cost of synthesizing unique peptides; require further validation with reaction monitoring	[72]
RP composition/stoichiometry	reaction monitoring	SRM		finding proper synthetic peptides; cost of synthesizing heavy peptides; limited in ion resolution of peptides	[66]
		PRM	increased ion resolution	finding proper synthetic peptides; cost of synthesizing heavy peptides	[67]
	bioinformatics	dripARF	fast hypothesis testing of ribosome heterogeneity; large-scale multi-study analysis	requires properly resolved ribosome structures; protocol differences; lack of directionality of the changes observed	[71]
RP modifications	enrichment for phosphopeptides + MS	TiO_2_-based MOAC	increases rate of detecting phosphorylated RPs; reproducible and scalable	metal ion contamination of phosphopeptides; lower peptide recovery	[82]
		IMAC	forms more stable bonds; more sensitive and specific	metal ion contamination of phosphopeptides	
		antibody-based enrichment	high recovery	affected by sequence motif	

^a^ dripARF, Differential RP Incorporation Prediction by Analysis of rRNA Fragments; ELIGOS, Epitranscriptional Landscape Inferring from Glitches of ONT Signals; GATK, Genome Analysis Toolkit; IMAC, Immobilized Metal Affinity Chromatography; iTRAQ, isobaric Tag for Relative and Absolute Quantification; MOAC, Metal Oxide Affinity Chromatography; PRM, Parallel Reaction Monitoring; PSI, Pseudouridine Site Identification; SRM, Selected Reaction Monitoring; TiO_2_, titanium dioxide; TMT, Tandem Mass Tags; rRNA, ribosomal ribonucleic acid; rDNA, ribosomal deoxyribonucleic acid; MS, mass spectrometry.

It is worth mentioning that these methods require dedicated training to implement. Different sophisticated protocols implementing these techniques, coupled with the bioinformatic skills to perform dedicated data analyses, attest to the need for dedicated training. Fortunately, computational tools exist as a possible alternative. Some of these tools reported in MS analysis tend to be premium software by biotechnology companies, requiring the need for the development of more simple, robust and open-source analysis pipelines and software. Again, limitations on computational tools also exist. Apart from limitations in data analysis, poor data quality or even the lack of desired data type could also influence the significance of these tools. Fortunately, open data are becoming the norm, yet still, exceptions in this category also exist.

Most methods require the purification of ribosomes as a starting point of the protocol. In most ribosome heterogeneity studies, sucrose gradient centrifugation was used to separate the different subunits of the ribosome, the monosome and the polysomes. Resolution of the sucrose gradient centrifugation is limited, and every fraction of ribosomes is therefore composed of varying depths of ribosome sub-populations [59]. So, when fractions are isolated and analysed, the results could be biased by a specific subpopulation of ribosomes overshadowing the others. Therefore, there is a need to develop tools that increase the resolution of identifying different sub-populations that occur.

In addition to the purification problem, issues could arise from subsequent processing of these fractions. The usage of synthetic tags on ribosomes to isolate certain sub-populations is a commonality in the field of studying the biological function of these sub-populations. However, considering the small size of RPs, adding these tags could potentially induce conformational changes in the RP. As a consequence of the alteration, structural changes can occur on the level of the whole ribosome. In essence, a small tag could potentially alter biological function [18]. Thus, when thinking of adding such tags to RPs, the possibility of structural and functional changes induced needs to be considered.

The field of ribosome heterogeneity is a rapidly growing one, but it is not without controversy [87]. Although evidence exists for the occurrence of heterogeneous ribosomes tied to different phenotypes including cancer [13] and ribosomopathies [18], additional studies are needed to bolster and understand how these modifications are regulated and how they come together to drive different phenotypes. To be able to achieve this requires the development of tools to accurately identify and quantify ribosome sub-populations. In the coming years we expect the field to increase in size, and as a result, the techniques detailed here to be used more often, and further developed for specific applications. It is also likely that both technology and, in particular, computational analysis will advance significantly, and it is obviously crucial that datasets and quality controls are standardized, to ensure reliable analysis.

The goal is to provide a clearer view of the diversity in ribosome populations, through parallelized detection and quantification of changes. Advances in multiplexing protocols, and more efficient and accurate algorithms, will help with this; however, benchmarked datasets are clearly needed to assess new informatic tools. Additionally, new algorithms and models are needed to improve the recovery of multiple rRNA modifications, especially in long-read sequencing approaches. In time, streamlined Ribo-Seq and proteomics pipelines, along with advances in the associated informatics approaches, will improve sensitivity, specificity and reproducibility in detecting and quantifying heterogeneous ribosomes.

## Data Availability

This article has no additional data.
